# Nutritional Recommendations for Cardiovascular Disease Prevention

**DOI:** 10.3390/nu5093646

**Published:** 2013-09-17

**Authors:** Sigal Eilat-Adar, Tali Sinai, Chaim Yosefy, Yaakov Henkin

**Affiliations:** 1 Zinman College for Physical Education & Sports, Wingate Institute, Netanya 42902, Israel; 2 School of Nutritional Sciences, Institute of Biochemistry, Food Science and Nutrition, The Robert H. Smith Faculty of Agriculture, Food and Environment, The Hebrew University of Jerusalem, Rehovot 76100, Israel; E-Mail: Tali.sinai@mail.huji.ac.il; 3 Cardiology Department, Barzilai Medical Center Campus, Ashkelon 78000, Israel; E-Mail: yosefy@barzi.health.gov.il; 4 Ben-Gurion University of the Negev, Beer Sheva 84105, Israel; E-Mail: yaakovh@bgu.ac.il; 5 Cardiology Department, Soroka University Medical Center, Beer-Sheva 84101, Israel

**Keywords:** guidelines, nutrition, cardiovascular, prevention

## Abstract

Lifestyle factors, including nutrition, play an important role in the etiology of Cardiovascular Disease (CVD). This position paper, written by collaboration between the Israel Heart Association and the Israel Dietetic Association, summarizes the current, preferably latest, literature on the association of nutrition and CVD with emphasis on the level of evidence and practical recommendations. The nutritional information is divided into three main sections: dietary patterns, individual food items, and nutritional supplements. The dietary patterns reviewed include low carbohydrate diet, low-fat diet, Mediterranean diet, and the DASH diet. Foods reviewed in the second section include: whole grains and dietary fiber, vegetables and fruits, nuts, soy, dairy products, alcoholic drinks, coffee and caffeine, tea, chocolate, garlic, and eggs. Supplements reviewed in the third section include salt and sodium, omega-3 and fish oil, phytosterols, antioxidants, vitamin D, magnesium, homocysteine-reducing agents, and coenzyme Q10.

## 1. Introduction

Lifestyle factors, including nutrition, play an important role in the etiology of Cardiovascular Disease (CVD). This position paper is written by collaboration of the Israel Heart Association and the Israel Dietetic Association.

We conducted a comprehensive literature search through electronic databases up to December 2012. We systematically searched published meta-analysis of intervention or cohort prospective studies that investigated the association between the relevant keywords of the chapter topic and cardiovascular health outcomes in electronic databases: The Cochrane Library (source: The Cochrane Central Register of Controlled Trials, Pubmed and Google Scholar. When multiple articles for a single study were present, we used the latest publication the most complete one. If needed, general historical information was added.

“If there were not enough data on cardiovascular morbidity or mortality (‘hard CV end points’), we searched for a possible influence on dyslipidemia or CVD risk factors (such as in the DASH diet)”. As nutritional data has limited “hard endpoint” data, especially from randomized trials, we needed to categorize some of the data based on surrogate endpoints as well.

The data were summarized literature with emphasis on the level of evidence (Table 1) and practical recommendations (Table 2) [1].

**Table 1:** Levels of evidence.

A	Data derived from multiple randomized clinical trials or meta-analyses
B	Data derived from a single randomized clinical trial or large non-randomized studies
C	Consensus of opinion of the experts and/or small studies, retrospective studies, registries

**Table 2:** Strength of statement and/or recommendation.

Class of recommendation	Definition	Suggested wording to use
Class I	Evidence and/or general agreement that a given statement and/or recommendation is beneficial	It is recommended/is indicated
Class II	Conflicting evidence and/or a divergence of opinion about the usefulness/efficacy of the statement and/or recommendation	
Class IIa	Weight of evidence/opinion is in favor of usefulness/efficacy	Should be considered
Class IIb	Usefulness/efficacy is less well established by evidence/opinion	May be considered
Class III	Evidence or general agreement that the treatment is not useful/effective and, in some cases, may be harmful	It is not recommended

Once the document has been finalized and approved by all the experts involved in the committee, it was submitted to outside specialists from the Israeli Heart Society and Israeli Dietetic Association for review.

The nutritional information is divided into three main sections: dietary patterns, individual food items, and nutritional supplements. The dietary patterns reviewed include low carbohydrate diet, low-fat diet, Mediterranean diet, and the DASH diet. Foods reviewed in the second section include: whole grains and dietary fiber, vegetables and fruits, nuts, soy, dairy products, alcoholic drinks, coffee and caffeine, tea, chocolate, garlic, and eggs. Supplements reviewed in the third section include salt and sodium, omega-3 and fish oil, phytosterols, antioxidants, vitamin D, magnesium, homocysteine-reducing agents, and coenzyme Q10.

## 2. Dietary Patterns

### 2.1. Low-Fat Diets

The consumption of a lower fat diet is generally accepted in all clinical guidelines on CV prevention, and will therefore not be discussed in detail in this manuscript. Briefly, the diet is based on total fat consumption of 25%–35% of total calories, of which, saturated fat (SFA) should be no more than 7%–10%, trans fat (TFA) less than 1%, unsaturated fats, mainly monounsaturated fats (MUFA) and omega-3 polyunsaturated fat (*n*-3 PUFA) should represent the rest of the calories from fat and cholesterol, for a total of less than 300 mg/day [2]. These recommendations can be achieved by choosing low-fat meats and emphasizing vegetables, low-fat dairy products and 1% milk, and lowering food containing TFA [3]. Generally, this diet increases the carbohydrate intake, and controversy remains about the type and amount of carbohydrate consumed [4].

### 2.2. Low-Carbohydrate Diets

A low-carbohydrate diet is defined as consumption of 30–130 g of carbohydrate per day or up to 45% of total calories [5]. Intervention studies resulted in a reduction in triglycerides (TG) and increase in HDL-cholesterol (HDL-C) [6]. The most recent systematic [7] review and meta-analysis among 1141 obese patients, showed the low-carbohydrate diets to be associated with significant decreases in body weight (−7.04 kg (95% CI −7.20/−6.88)), body mass index (BMI) (−2.09 kg/m^2^) (95% CI −2.15/−2.04), systolic blood pressure (−4.81 mmHg (95% CI −5.33/−4.29)), diastolic blood pressure (−3.10 mmHg (95% CI −3.45/−2.74)), plasma TG (−29.71 mg/dL (95% CI −31.99/−27.44)), as well as an increase in HDL-C (1.73 mg/dL) [95% CI 1.44/2.01]. Low-density lipoprotein cholesterol (LDL-C) and creatinine did not change significantly. The authors concluded that low-carbohydrate diets result in favorable effects on body weight and major CV risk factors; however, the effects on long-term health are unknown. A two-year Dietary Intervention Randomized Controlled (DIRECT) trial among 322 moderately obese participants that compared low-fat, Mediterranean, and low-carbohydrate diets found that compared to the other diets, the low-carbohydrate diet was most effective in weight loss, decreasing TG and increasing HDL-C levels [8]. However, at follow-up four years after completion of the randomized study, the weight regain in the low-carbohydrate group was also most prominent, resulting in similar overall weight loss between the low-fat and low-carbohydrate groups. Despite this partial weight regain, there was a reduction in the ratio of LDL-C to HDL-C (a reduction of 0.16, *p* = 0.04), and the reduction in TG levels (11.3 mg/dL, *p* = 0.02) remained significant in the low-carbohydrate group, suggesting a long-lasting, favorable post-intervention effect.

### 2.3. Mediterranean Diet

The Mediterranean diet was originally described in Crete and Italy, and is characterized by a relatively high fat intake (40%–50% of total daily calories), of which SFA comprises ≤8% and MUFA 15%–25% of calories. It is characterized by a high omega-3 fatty acid intake from fish and plant sources and a low Omega-6:Omega-3 ratio of 2:1–1:1 compared to 14:1 in Europe [9,10]. It is based on seasonal, local, fresh vegetables, fruits, whole bread and grains, legumes, nuts, and olive oil. Moderate intake of dairy products (low-fat), as well as eggs, fish, and chicken are allowed, while red meat is avoided. Small to moderate quantities of wine are encouraged with meals [8]. Adherence to the Mediterranean diet was associated with a low risk of coronary heart disease (CHD), as shown in a meta-analysis of seven cohort studies; a 2-point increase in adherence to the Mediterranean diet was associated with a significant reduction of overall mortality. RR = 0.92; [95% CI 0.90–0.94], CV incidence or mortality (RR = 0.90; (95% CI 0.87–0.93)) [11]. In a multicenter random intervention trial in Spain, participants who were at high cardiovascular risk, but with no cardiovascular disease at enrollment, were divided to one of three diets: a Mediterranean diet supplemented with extra-virgin olive oil, a Mediterranean diet supplemented with mixed nuts, or a control diet (advice to reduce dietary fat). The primary end point was the rate of major cardiovascular events (myocardial infarction, stroke, or death from cardiovascular causes). On the basis of the results of an interim analysis, the trial was stopped after a median follow-up of 4.8 years.

The multivariable-adjusted HR were: HR = 0.70 (95% CI 0.54–0.92) and 0.72 (95% CI, 0.54–0.96) for the group assigned to a Mediterranean diet with extra-virgin olive oil (96 events) and the group assigned to a Mediterranean diet with nuts (83 events), respectively, versus the control group (109 events). No diet-related adverse effects were reported. This study confirmed that, among persons at high cardiovascular risk, a Mediterranean diet supplemented with extra-virgin olive oil or nuts reduced the incidence of major cardiovascular events [12].

### 2.4. Dash Diet

The Dietary Approach to Stop Hypertension (DASH) diet is a nutritional program assembled in the 1990s and assessed in intervention controlled trials. Its main target was to lower blood pressure, and therefore CVD incidence, by nutritional means. The DASH diet comprises vegetables and fruits, as well as low-fat dairy products, whole grains, chicken, fish, and nuts. On the other hand it is low in fat, meat, sweets, and sodas. The DASH diet, summarized in Table 3, provides more calcium, potassium, magnesium, and dietary fiber and less fat, SFA, cholesterol, and sodium than the typical western diet [13].

**Table 3:** Dietary Approach to Stop Hypertension (DASH) diet composition [13].

Nutrient	Daily quantity
Total fat	27% of total calories
SFA	6% of total calories
Carbohydrates	55% of total calories
Protein	18% of total calories
Cholesterol	150 mg
Fiber	31 g
Potassium	4700 mg
Magnesium	500 mg
Calcium	1240 mg

Recommendations and level of recommendations of dietary patterns are summarized in Table 4.

**Table 4:** Level of evidence and classes of recommendations for food patterns.

Food pattern	Recommendations	Strength	Level of evidence
**Low-fat diet**	Low-fat diet with restricted calories may present a healthy alternative to the typical Western diet. It may improve quality and life expectancy in healthy people, as well as in patients with overweight, diabetes, and CVD.	II a	**A**
**Low-carbohydrate Diet**	In the short-run, low-carbohydrate diets lead to a greater weight loss compared to low-fat diets. Some studies have shown that this advantage is retained at 2 years but not at longer follow-up periods	II b	A
	Low-carbohydrate diets are preferable to a low-fat diet in reducing TG levels and increasing HDL-C blood levels. It should be emphasized that carbohydrates should preferably be replaced by unsaturated vegetable fats.	II a	A
	Low-carbohydrate diets, which include 30%–40% of calories from carbohydrates and are low in saturated fat and high in monounsaturated fat, were found to be safe in healthy and overweight individuals at follow-up up to 4 years.	II a	A
**Mediterranean Diet**	A Mediterranean diet with restricted calories may present a healthy alternative to the typical Western diet. It may improve quality and life expectancy in healthy people, as well as in patients with overweight, diabetes, and CVD.	II a	A
	Mediterranean diets are preferable to a low-fat diet in reducing TG levels, increasing HDL-C blood levels, and improving insulin sensitivity.	II a	A
**DASH Diet**	The DASH diet is recommended to prevent hypertension and lower blood pressure.	I	A
	The diet should be accompanied by lifestyle changes such as: weight reduction in overweight people, increased physical activity, sodium restriction, and alcohol avoidance.	I	A

Compared to the typical western diet, the DASH diet reduced systolic and diastolic blood pressure by 11.4 and 5.5 mmHg, respectively, and by 7.2 and 2.8 mmHg, respectively, in patients with hypertension (HTN). The blood pressure decrease was observed in normotensive participants as well [14,15]. Adding sodium restriction to the DASH diet further reduced the blood pressure [16]. It also improved autonomic and vascular function and lowered left ventricular mass among overweight patients with HTN. This influence was most prominent when accompanied by weight reduction and increased physical activity [17]. The PREMIER trial combined the DASH diet with a lifestyle program aimed at reducing overweight, increasing physical activity, and restricting sodium and alcohol intake. In patients with HTN, systolic and diastolic blood pressures were reduced by 14.2 and 7.4 mmHg, respectively. A decrease in blood pressure was observed in normotensive participants as well [15]. Based on these data, the theoretical decrease in Framingham risk score for CHD was 12% greater when adding lifestyle changes to the DASH diet [18].

### 2.5. Conclusions

All four dietary patterns described above are useful for reducing CVD risk factors, and some have also shown a favorable effect on plaque regression [19] and CVD mortality [16]. Thus, every patient should adopt a dietary approach that conforms to his or her personal preferences; however, the long-term effects of some of these diets, and especially a high saturated-fat, low-carbohydrate diet, on CVD and total mortality have not been fully assessed.

## 3. Individual Food Items

### 3.1. Whole Grains and Dietary Fiber

Whole grains represent unprocessed grains that contain the endosperm; the bran (the outer layer of the whole grain) and the germ are in the same relative proportions as they exist in the intact grain. In contrast, refined grains retain only the endosperm. Common whole grains include: whole wheat, whole rice, barley, corn, rye, oats, millet, sorghum, teff, triticale, canary seed, Job’s tears, fonio, and wild rice [20].

Dietary fiber consists of the remnants of edible plant cell polysaccharides, lignin, and associated substances resistant to hydrolytic digestion by the human alimentary enzymes [21]. They can be divided into: insoluble fiber, which includes cellulose and lignin, and is found in vegetables, some fruits, and whole grains (including the wheat germ); and soluble fiber, which includes fruits, pectin, guar gum, and mucilage. Soluble fiber is found in legumes and in oat bran [22]. In a Cochrane review, 10 studies of 4–8 weeks duration that included 56–85 g of fiber in individuals with CHD or CHD risk factors were reviewed. Eating whole grains decreases total cholesterol levels by 7.7 mg/dL (95% CI 3.9–12) and LDL-C levels by 6.9 mg/dL (95% CI 3.5–10.8) [23]. In a meta-analysis of 67 controlled intervention trials, daily consumption of 2–10 g/day soluble fiber (mainly beta-glucan, psyllium, and pectin) lowered LDL-C by 2.2 mg/dL (95% CI 1.7–2.7) with no significant changes in HDL-C or triglycerides (TG) [24]. 

The American Heart Association (AHA) [3], The American Dietetic Association [25] and the National Cholesterol Education Program (ATP III) [26] guidelines include a recommendation to increase dietary soluble fiber intake. The question of whether added fiber used as a food supplement can similarly protect against CVD is still controversial. Despite this, the Food and Drug Administration (FDA) approved a health claim on soluble fiber from whole oats, whole grain barley products, and barley beta fiber [27]. The DRI recommends consumption of 14 g dietary fiber per 1000 kcal, or 25 g for adult women and 38 g for adult men [22].

### 3.2. Vegetables and Fruits

Although the botanic term “fruit” refers to the seeds and surrounding tissues of a plant, the foods that are commonly referred to as “fruits” for culinary purposes are pulpy seeded tissues that have a sweet (oranges, apples, pears, blueberries) or tart (lemons, limes, cranberries) taste. By culinary definition, “vegetables” are edible plant parts including stems and stalks (celery), roots (carrots), tubers (potatoes), bulbs (onions), leaves (spinach, lettuce), flowers (artichokes), some fruits (cucumbers, pumpkin, tomatoes), and seeds (beans, peas). Vegetables are in general less sweet or tart than fruits [28].

The evidence that vegetables and fruits are associated with reduced CHD risk is based only on epidemiological data. In a meta-analysis of nine cohort studies (including 91,379 men, 129,701 women, and 5007 CHD events), CHD risk was lower by 7% for each additional fruit serving a day (RR 0.93, 95% CI 0.89–0.96; *p* < 0.001) [29]. The association between vegetable intake and CHD risk was heterogeneous and more marked for CV mortality (0.74, 95% CI 0.75–0.84; *p* < 0.0001) than for fatal and nonfatal myocardial infarction (0.95, 95% CI 0.92–0.99; *p* < 0.006).

There are no interventional studies that specifically evaluated the influence of vegetables and fruits on CHD risk. In interventional studies where vegetable and fruit consumption was part of the nutritional recommendations, CHD risk reduction was documented [10,11]. Vegetable and fruit consumption was associated with lower blood pressure [13,14,15,18], but the association with other CHD risk factors is not clear. Despite the lack of intervention studies, the American Heart Association (AHA) recommends intake of at least 8 vegetables and fruits a day [3].

The mechanism of action is not known, but it is assumed that the healthy effect of vegetables and fruits can be attributed to the dietary fiber and antioxidants in these food items [30]. Vegetables and fruits also act as a low-calorie, low-sodium, and satiating food.

### 3.3. Nuts

Nuts (tree nuts and peanuts) are nutrient-dense foods with complex matrices rich in unsaturated fatty acids and other bioactive compounds: high-quality vegetable protein, fiber, minerals, tocopherols, phytosterols, and phenolic compounds [31]. By definition, tree nuts are dry fruits with one seed in which the ovary wall becomes hard at maturity. This group includes almonds, hazelnuts, walnuts, pistachios, pine nuts, cashews, pecans, macadamias, and Brazil nuts. The consumer definition also includes peanuts, which botanically are groundnuts or legumes but are widely identified as part of the nuts food group. In addition, peanuts have a nutrient profile similar to that of tree nuts. Although chestnuts are tree nuts as well, they are different from all other common nuts because of being starchier and having a different nutrient profile [32,33,34].

Epidemiological data show a consistent negative association between nuts consumption and CHD risk [34]. Some of the studies found a dose-response pattern of association. An analysis of four studies from the United States concluded that high nut intake is associated with a 35% risk reduction for CVD [35].

A pooled analysis was done using data from 25 intervention nut consumption trials (including walnuts, almonds, macadamias, pecans, peanuts, and pistachios) conducted in seven countries among 583 men and women with normolipidemia and hypercholesterolemia who were not taking lipid-lowering medications. With a mean daily consumption of 67 g of nuts, LDL-C concentration was reduced by a mean of 10.2 mg/dL (−13.1 to −7.4 mg/dL, 7.4% reduction, *p* < 0.01), with no significant change in HDL-C levels. Mean TG levels were reduced by 20.6 mg/dL (−30.7 to −9.9 mg/dL, 10.2% reduction, *p* > 0.05) in subjects with blood triglyceride levels ≥150 mg/dL but not in those with normal TG levels. The effects of nut consumption were dose related. Different types of nuts had similar effects on blood lipid levels. The lipid-lowering effects were greatest among subjects with high baseline LDL-C and with low BMI [36]. However, there are no trials relating consumption to CVD endpoints.

#### Possible Mechanisms

The mechanism of action can be attributed to the high polyunsaturated fatty acids (PUFA) and low SFA content. Some nuts (such as walnuts) also contain alpha-linolenic fatty acid. Other macronutrients include plant protein and fiber; micronutrients including potassium, calcium, magnesium, and tocopherols; and phytochemicals such as phytosterols, phenolic compounds, resveratrol, and arginine [35]. Those nutrients may have a beneficial effect on blood lipids as well as other CHD risk factors such as oxidation and inflammation. It is also possible that the substitution of high SFA, sodium, and sugar food by nuts and almonds can also explain this positive effect.

### 3.4. Soy

Soy protein refers to the protein that is found in soybeans and is often used to replace animal protein in an individual’s diet. The soybean is a legume that contains no cholesterol and is low in saturated fat, and is the only vegetable food that contains all eight essential amino acids. Soybeans are also a good source of fiber, iron, calcium, zinc, and B vitamins [37]. Soy beans are the best known and most widely consumed food that contains phytoestrogen (isoflavones), which are plant components that interact with mammalian endocrine systems [38].

#### Intervention Studies

In 22 randomized trials, isolated soy protein with isoflavones was compared with casein or milk protein, wheat protein, or mixed animal proteins. The range of soy protein was 25 to 135 g/day; the range for isoflavones was 40 to 318 mg/day. LDL or non-HDL cholesterol concentrations decreased in most studies, statistically significantly in 8, with an overall effect of about 3% (weighted average). In a meta-analysis soy protein isolate, but not other soy products or components, significantly reduced diastolic blood pressure (9 studies, mean reduction 1.99 mmHg; 95% CI −2.86, −1.12) and LDL-C (39 studies, mean reduction 7.3 mg/dL; 95% CI −9.3, −5.4) [39].

Although the improvement in lipoproteins and blood pressure induced by soy protein is of small and questionable clinical significance, consumption of soy protein-rich foods may indirectly reduce CVD risk if it replaces animal products that contain saturated fat and cholesterol [3].

In October 1999, the FDA approved labeling for foods containing soy protein as protective against coronary heart disease. The FDA based this decision on clinical studies showing that at least 25 g of soy protein per day lowered total and LDL cholesterol. The FDA requires for the claim that a serving contain at least 6.25 g of soy protein, 25% of the necessary daily quantity of protein (25 g), with the expectation that foods containing soy protein would be eaten at least four times per day. The FDA also stated that “the evidence did not support a significant role for soy isoflavones in cholesterol-lowering effects of soy protein” [40]. However, caution should be exercised when extrapolating this recommendation to processed meats, which may include soy components, as a meta-analysis found a 42% higher risk of CHD (*n* = 5 studies; RR per 50 g serving per day = 1.42; 95% CI 1.07–1.89; *p* = 0.04) [41] in individuals consuming processed meats.

The hormonal effects of dietary soy and soy extracts were extensively evaluated. A meta-analysis of 178 studies revealed inconsistent effects on climacteric symptoms [40]. Another meta-analysis of prospective studies suggested that soy isoflavone intake is associated with a significant reduced risk of breast cancer incidence in Asian populations, but not in Western populations [42], although there was no dose-response relationship between total isoflavone intake and risk of breast cancer incidence.

### 3.5. Dairy Products

Dairy products are rich in minerals (calcium, potassium, and magnesium), protein (casein and whey), and vitamins (riboflavin and vitamin B-12) that can exert beneficial effects on CVD. On the other hand, the presence of saturated fat in dairy products causes concern over potential adverse CV effects [43].

There is conflicting evidence on the association between dairy intake and CVD. The number of cohort studies that give evidence on individual dairy food items is very small. However, a meta-analysis suggests a reduced risk in the subjects with the highest dairy consumption relative to those with the lowest intake: RR = 0.87 (95% CI 0.77–0.98) for all-cause deaths (6 studies), RR = 0.92 (95% CI 0.80–0.99) for ischemic heart disease (9 studies), and RR = 0.79 (95% CI 0.68–0.91) for stroke (13 studies) [44]. In another meta-analysis of 17 prospective studies, a modest inverse association was found between milk intake and risk of overall CVD (4 studies); RR = 0.94 per 200 mL/day )95% CI 0.89–0.99). Milk intake was not associated with risk of CHD, stroke, or total mortality. When stratified into high-fat and low-fat dairy products no significant associations were found with CHD [45].

#### 3.5.1. Possible Mechanisms

Suggested mechanisms for the blood-pressure lowering effects of dairy products include the high content of potassium, magnesium, and calcium. In the DASH diet, the combination diet rich in fruits, vegetables, and 2.7 servings per day of dairy products (predominantly low-fat), substantially lowered blood pressure [16]. An association between calcium intake and lower body weight and fat mass has been described [46]. There is some evidence that certain fermented products (especially by *Lactobacillus helveticus*) have a mildly decreasing effect on HTN, probably because of bioactive peptides [47]. The lack of effect of the high saturated fat content on LDL-C levels is attributed to the unique fatty acid composition of dairy products, consisting mostly of short-chain fatty acids and stearic acid.

#### 3.5.2. Conclusions

Despite the contribution of dairy products to the saturated fatty acid composition of the diet, and given the diversity of dairy foods of widely differing fat composition, there is no clear evidence that dairy food consumption is consistently associated with a higher risk of CVD [48] and some evidence that low-fat products may have beneficial effects on blood pressure.

The general health recommendation is to prefer low-fat products in order to reduce SFA intake. This recommendation is based on data from the Nurse’s Health Study, in which the high-fat to low-fat dairy consumption ratio was associated with significantly greater risk [49].

### 3.6. Alcoholic Drinks

The consumption of alcohol (ethanol) is widely accepted in many social situations. Most data on the association between alcohol and CVD come from short-term interventional studies on the effects of alcohol on risk factors as well as long-term observational mortality studies.

Based on cohort studies, the evidence suggests a J- or U-shaped relationship between alcohol consumption and risk of CHD [50]. In a meta-analysis of 84 prospective cohort studies, the pooled adjusted RR for moderate alcohol drinkers relative to non-drinkers was 0.75 (95% CI 0.70–0.80) for CVD mortality (21 studies), 0.71 (95% CI 0.66–0.77) for incident CHD (29 studies), and 0.75 (95% CI 0.68–0.81) for CHD mortality (31 studies) [51]. Moderate intake of alcoholic beverages (1 to 2 drinks per day) is associated with a reduced risk of CHD in healthy populations [52]. The findings do not implicate an advantage of one type of drink over another [53].

Among CVD patients, binge drinkers, defined as those who consumed 3 or more drinks within 1 to 2 h, had double the total and CV mortality risk of regular drinkers [54]. Episodic heavy alcohol drinking, but not moderate drinking, is reportedly associated with risk of atrial fibrillation [55]. A detrimental risk for heart disease is not reached when the average consumption is 20–72 g/day [56]. Excessive consumption is associated with a higher risk for alcohol abuse, hypertension, overweight, various malignancies, automobile accidents, trauma, and suicide [57].

#### 3.6.1. Possible Mechanisms

Numerous mechanisms have been proposed to explain the benefit of light-to-moderate alcohol intake on the heart, including an increase in HDL-C, reduction in plasma viscosity and fibrinogen concentration, increase in fibrinolysis, decrease in platelet aggregation, improvement in endothelial function, reduction in inflammation, and promotion of antioxidant effects [58,59]. However, despite the biological plausibility and observational data in this regard, these are still insufficient to prove causality. Daily intake of more than moderate amounts of alcoholic beverages can also be a risk factor for the development of HTN, increased plasma TG levels, can serve as a source of excess calories, as well as increased risk for breast and other cancers [60]. Patients who are hypertensive have high TG levels and women at high risk of breast cancer should avoid alcoholic beverages [58].

#### 3.6.2. Conclusions

Despite the evidence from cohort studies on the inverse association between moderate alcohol drinking and CVD, current guidelines do not recommend to begin consuming alcohol for preventing CVD. Individuals who regularly consume alcohol and who do not have a family history of cancer should do so in moderation—the equivalent of no more than one drink in women or two drinks in men per day (Table 5). Alcohol should be avoided in pregnant women [54]. People who intend to drive should avoid drinking alcohol.

**Table 5:** Energy content and ethanol in alcoholic beverages [61].

	Spirits	Wine	Beer
The drink portion size (mL)	45	150	350
Energy (kca/portion)	100	120–125	150
Ethanol (g/portion)	14–15	15	14

### 3.7. Coffee and Caffeine

Coffee is one of the most widely consumed beverages in the world. The remaining sources of caffeine include primarily tea, cocoa products, cola beverages, and “energy” drinks [62]. Caffeine (1,3,7-trimethylxanthine) is by far the best characterized compound in coffee. Coffee also contains chlorogenic acid, flavonoids, melanoidins, and various lipid-soluble compounds such as furans, pyrroles, anmaltol. Many of these compounds are efficiently absorbed, have relatively high bioavailability, and have been shown to have antioxidant properties. An estimated 80%–90% of adults report regular consumption of caffeine-containing beverages and foods, making it the most widely consumed stimulant in the world. There is a possible bias in comparing caffeinated and decaffeinated coffee. However, most epidemiologic studies do not distinguish former users of caffeinated coffee who may have switched to decaffeinated coffee because of a health problem, and never-users who may be avoiding caffeine as part of a healthy lifestyle [63]. Energy content and ethanol in alcoholic beverages are summarized in Table 6.

**Table 6:** Caffeine content in selected food and drink products.

Product	Quantity	Caffeine content (mg)
Coffee, instant	1 glass, 190 mL	75
Roasted, ground, perculated or filter, or espresso	1 glass, 190 mL	100–180
Coffee, decaffeinated	1 glass, 190 mL	4
Tea, green	1 glass, 190 mL	24
Tea, black	1 glass, 190 mL	15–24
Tea, leaf or bag	1 glass, 190 mL	40–100
Cocoa drink	1 glass, 200 mL	1.1–8.2
Energy drinks containing caffeine or Guarana	1 can, 250 mL	28–87
Coca Cola (regular, diet)	1 can, 330 mL	42 (10–70)
Chocolate	50 g	6–40

Based on date from: Israeli Health Ministry position paper and from [61].

Coffee consumption has long been suspected of being a contributing factor in the development of CVD, based mainly on case-control studies [63,64]. However, in the last few years there are accumulated data suggesting no harm [65,66,67], and even a protective association between moderate coffee drinking and CHD morbidity and CVD mortality [68,69]. Lately, the risk for developing type 2 diabetes was found to be lower in individuals who consumed four or more cups of coffee per day compared with those who drank less than two cups per day [70].

#### 3.7.1. Possible Mechanisms

Several mechanisms have been proposed to explain the harmful as well as protective effects that certain components of coffee may have on the development of CHD. These include the effects of coffee on blood pressure, serum cholesterol and homocysteine levels, oxidation, and inflammation [65].

#### 3.7.2. Conclusions

Although regular consumption of moderate quantities of coffee seems to be associated with a small protection against CAD, results from randomized clinical trials about its beneficial effects are lacking. At present, for adults consuming moderate amounts of coffee (3–4 cups/day providing 300–400 mg of caffeine), there is little evidence of health risks and some evidence of health benefits [66]. However, some groups, including people with HTN, children, adolescents, and the elderly, may be more vulnerable to the adverse effects of caffeine. In addition, currently available evidence suggests that it may be prudent for pregnant women to limit coffee consumption to 3 cups/day providing no more than 300 mg/day of caffeine [71]. Fatal or life-threatening caffeine overdoses generally involve the ingestion of caffeine-containing medications. Oral doses of 5–50 g (mean 10 g) have resulted in fatalities in adults, and the lethal dose is estimated at 100–200 mg/kg of body weight. Ingestion of 15–30 mg/kg has resulted in significant toxicity. Symptoms of caffeine overdose may include agitation, delirium, seizures, dyspnea, cardiac arrhythmia, myoclonus, nausea, vomiting, hyperglycemia, and hypokalemia [72].

### 3.8. Tea

Tea has been one of the most popular beverages for 4000 years. Brewed from the plant *Camellia sinensis*, tea is consumed in different parts of the world as green, black, or Oolong tea. Of the tea produced worldwide, 78% is black tea, which is usually consumed in the Western countries; 20% is green tea, which is commonly consumed in Asian countries (mainly Japan and China); and 2% is Oolong tea, which is produced (by partial fermentation) mainly in southern China. Green and black teas are processed differently during manufacturing. To produce green tea, freshly harvested leaves are steamed, yielding a dry, stable product. A typical tea beverage, prepared in a proportion of 1 g leaf to 100 mL water in a 3 min brew, usually contains 250–350 mg tea solids, comprising 30%–42% catechins and 3%–6% caffeine [72].

#### 3.8.1. Possible Mechanisms

Most of the beneficial effects of tea are attributed to its polyphenolic flavonoids, known as catechins. The major flavonoid is epigallocatechin-3-gallate (EGCG). These polyphenols account for up to 40% of the dry weight of green tea, and purified EGCG has been the focus of research in recent years [73].

#### 3.8.2. Observational Studies

A population-based prospective cohort study (the Ohsaki Study) included 40,530 persons in Miyagi prefecture in northern Japan [74]. Risk for CVD mortality was found with increasing green tea consumption (occasional, 1–2 cups/day, 3–4 cups/day, and 5 or more cups/day, when the volume of a typical cup of green tea is 100 mL) was: 1.00, 0.84 (95% CI 0.63–1.12), 0.69 (95% CI 0.52–0.93), 0.69 (95% CI 0.53–0.90), respectively (*p* for trend = 0.004). Within CVD mortality, the stronger inverse association was observed for stroke mortality. A meta-analysis of 18 studies included 13 studies on black tea and 5 studies on green tea. For black tea, no significant association was seen with the risk for developing CAD. For green tea an increase of 1 cup/day was associated with a 10% decreased risk of CAD incidence (RR: 0.90, 95% CI: 0.82–0.99) [75]. In a meta-analysis of 194,965 participants in nine studies, individuals consuming ≥3 cups of tea per day had a 21% lower risk of stroke than those consuming <1 cup per day (absolute risk reduction, 0.79, 95% CI 0.73–0.85) [76].

#### 3.8.3. Intervention Studies

No randomized controlled trial studied the effects of tea consumption on CVD morbidity or mortality; however, many studies evaluated the effects of tea on CV risk factors. More than half of the randomized controlled trials have demonstrated the beneficial effects of green tea on CVD risk profiles. These results suggest a plausible mechanism for the beneficial effects of green tea [75].

In a meta-analysis of 133 trials, black tea consumption increased systolic (5.69 mmHg; 95% CI 1.52–9.86; 4 studies) and diastolic (2.56 mmHg; 95% CI 1.03–4.10; 4 studies) blood pressure, but chronic consumption did not appear to affect blood pressure. Green tea did not appear to affect blood pressure, but reduced LDL cholesterol levels (−9 mg/dL; 95% CI −4.6, −13.1; 4 studies) [39]. Other suggested mediators for the association between tea consumption and reduced CVD risks include anti-inflammatory, anti-oxidant, and anti-proliferative effects, as well as favorable effects on endothelial function [77].

#### 3.8.4. Adverse Effects

There do not appear to be any significant side-effects or toxicity associated with green tea consumption. In general, the stimulatory effect from green tea is considerably less than that from coffee [78]. However, tea extract may cause gastrointestinal irritation. Although there are a few case reports of liver toxicity resulting from the ingestion of large quantities of green tea or green tea extract, the incidence of this potential adverse effect appears extremely low. Since green tea may interfere with the absorption of iron supplements, iron supplements should not be ingested together with green tea components. Possible interactions between green tea and other medications have also been reported [79].

### 3.9. Chocolate

Cocoa is rich in polyphenols, similar to those found in green tea. Chocolate and cocoa are two different things. Cocoa is the non-fat component of cocoa liquor (finely ground cocoa beans) that is used in chocolate making or as cocoa powder (commonly 12% fat) for cooking and drinks [80]. Fat and sugar are major components of chocolate, which has high caloric content that needs to be taken into account when assessing possible risks and benefits of recommending chocolate consumption for health purposes. However, the major fatty acids in chocolate are oleic, palmitic, and stearic acids; oleic and stearic acids may have a neutral effect on blood lipid levels [81]. Chocolate, especially of the milk variety, contains large amounts of sugar and has possible implications for dental health and diabetes if eaten in large quantities, although carbohydrates might play a role in improving uptake of polyphenols. Cocoa itself is much easier to recommend on a health basis as it is not high in sugar and fat.

#### 3.9.1. Observation Studies

A recent meta-analysis of seven observational studies reported a beneficial association between higher levels of chocolate consumption and the risk of CVD. The highest levels of chocolate consumption were associated with an adjusted lower risk for CVD (RR = 0.63 (95% CI 0.44–0.90) and a 29% reduced risk stroke compared with the lowest levels [82]. However, most of the studies did not adjust for socioeconomic factors, which may confound this association.

#### 3.9.2. Intervention Studies and Possible Mechanisms

Most of the existing evidence is on intermediate factors of CVD. Recent studies (both experimental and observational) have suggested that chocolate consumption has a positive influence on human health, with antioxidant, antihypertensive, anti-inflammatory, anti-atherogenic, and anti-thrombotic effects as well as influence on insulin sensitivity, vascular endothelial function, and activation of nitric oxide [82]. Dietary flavanols have also been shown to improve endothelial function and to lower blood pressure by causing vasodilation in the peripheral vasculature and in the brain [83].

Despite this array of benefits, there is a lack of well-designed clinical studies demonstrating a CV benefit of chocolate. The high caloric content of chocolate, particularly of some less pure forms, should be considered before recommending uncontrolled consumption [84].

### 3.10. Garlic

The bulk of the dry weight of garlic (*Allium sativum*) contains mainly fructose-containing carbohydrates, followed by sulfur compounds, protein, fiber, and free amino acids. It also contains high levels of saponins, a variety of minerals and vitamins A and C, and a high phenolic content. Garlic has been attributed with favorable CV effects due to its high content of thiosulfinates, including allicin, which is considered to be the active component of garlic. Allicin is formed when alliin, a sulfur-containing amino acid, comes into contact with the enzyme alliinase when raw garlic is chopped, crushed, or chewed. Over the years, different garlic preparations have been investigated for their prevention and treatment of CV disease, including raw garlic, garlic powder tablets, oil of steam-distilled garlic, oil of oil-macerated garlic, ether-extracted oil of garlic, and aged garlic extract. All these preparations differ in their composition, which complicates comparison of studies [85]. Dried garlic preparations containing alliin and alliinase must be enteric coated to be effective because stomach acid inhibits alliinase. Because alliinase also is deactivated by heat, cooked garlic is less powerful medicinally [86].

Long-term observation studies are missing. Intervention trials focused on CVD risk factors.

In a meta-analysis of 29 trials garlic was found to significantly reduce total cholesterol (−0.3, 95% CI –2.3, –12.7 mg/dL) but exhibited no significant effect on LDL-C or HDL-C levels [87]. However, in a later meta-analysis of 13 trials there was no significant difference in effects on all outcome measures examined when compared with placebo [88]. A review of trials assessing the effect of garlic on thrombotic risk showed modest but significant decreases in platelet aggregation with garlic compared with placebo [89]. The antihypertensive effects of garlic have been studied but remain controversial [88].

#### 3.10.1. Adverse Effects

Proven adverse effects include malodorous breath and body odor. Other unproven effects included flatulence, esophageal and abdominal pain, allergic reactions, and bleeding [86].

#### 3.10.2. Dosage

The effective dose of garlic has not been determined. Dosages generally recommended in the literature for adults are 4 g (one to two cloves) of raw garlic per day, one 300 mg dried garlic powder tablet (standardized to 1.3 percent alliin or 0.6 percent allicin yield) two to three times per day, or 7.2 g of aged garlic extract per day [86].

### 3.11. Eggs

During the past 40 years, the public had been warned against frequent egg consumption due to the high cholesterol content in eggs and the potential association with CVD [90]. This was based on the assumption that high dietary cholesterol consumption is associated with high blood cholesterol levels and CVD. However, subsequent research suggests that, in contrast to SFA and TFA, dietary cholesterol in general and cholesterol in eggs in particular have limited effects on the blood cholesterol level and on CVD [91]. Eggs are also a source for high biological value protein, as well as vitamins and minerals such as folic acid, vitamin B12, vitamins E and D, selenium, choline, zinc, *etc*. About 50% of the fat in the egg is MUFA [92].

Level of evidence and classes of recommendations for food items is summarized in Table 7.

**Table 7:** Level of evidence and classes of recommendations for food items.

Food item	Recommendations	Strength	Level of evidence
Whole grains and dietary fiber	The recommended dietary fiber intake is 14 g per 1000 kcal, or 25 g for adult women and 38 g for adult men.	II a	B
	It is recommended to increase dietary fiber intake in order to reduce blood LDL-C and glucose.	I	A
Vegetables and fruits	It is recommended to consume at least 8 portions of vegetables and fruits a day. Preferably root vegetables and deep-colored fruits such as spinach, carrot, peach, and blueberries (since they usually contain more micronutrients compared to other vegetables and fruits).	II a	B
	It is recommended to eat the whole fruit rather than fruit juice because of the fiber content and the satiation.	II a	A
	It is recommended to use cooking techniques such as sautéing or simmering that preserve the micronutrients in the vegetables and fruits without additional calories, SFA, TFA, salt or sugar.	II a	A
	In cases of disease influenced by dietary carbohydrates, sodium, or potassium (diabetes, kidney, coagulation), vegetables and fruits quantity should be personally adjusted.	II a	A
Nuts and almonds	It is recommended to consume 20–30 g/day of unsalted nuts and almonds, or 150 g/week, as a substitute for other food (with equal caloric content to prevent weight gain) in order to		
	improve blood lipids.	II a	A
	Reduce CVD risk.	II a	B
Milk and dairy products	It is recommended to include dairy products (preferably low-fat and without added sugar) as part of a balanced diet.	II a	B
	Low-fat milk and dairy products lower blood pressure.	I	A
	There is epidemiologic data to suggest an association between dairy product consumption and reduced CVD.	II a	B
	At this stage, there is no evidence that calcium and/or vitamin D supplements prevent CVD (supplements may be taken for other indications such as osteoporosis).	III	C
Alcohol	Due to the absence of interventional controlled studies of moderate alcohol consumption with clinical endpoints, there is no recommendation to start drinking alcohol for health benefits.	III	C
	In individuals who regularly drink a moderate amount of alcohol (1 drink a day in women and 2 drinks per day in men) with meals, there is an associated reduced CVD incidence. Larger amounts should be discouraged.	I	B
	Individuals with liver disease and/or fatty liver, HTN, or hypertriglyceridemia, and pregnant women should avoid alcohol consumption.	III	B
	Alcohol drinking should be avoided before driving and/or coordinated activity.	III	A
Coffee	In order not to increase CVD morbidity and mortality, and/or side effects, it is recommended to consume the following amounts of caffeine: Healthy adults without caffeine sensitivity: up to 400 mg/day; Pregnant women: up to 200–300 mg/day; Children 4–6 years old: up to 45 mg/day.	II a	B
Green tea	Green tea consumption is associated with a lower risk for stroke and CVD. However, the causal effect and the dose needed for this effect is unknown.	II a	B
	Green tea consumption may reduce LDL-C; however, the effect-size and the dose needed are still unknown.	II b	A
Chocolate	It is not recommended to consume chocolate for CVD prevention.	III	C
	Dark chocolate, with a high cocoa percent, has abundant antioxidants and therefore preferable over milk chocolate.	II a	B
Garlic	Eating 2 garlic cloves a day may marginally reduce blood cholesterol levels.	II b	C
Eggs	Consumption of 5 eggs per week does not significantly increase CVD risk in healthy people.	II a	B
	In people with diabetes, CHD, and/or hypercholesterolemia that is not medically balanced there may be an increased risk from egg consumption. It is recommended to limit egg consumption to 3–4 per week, including eggs contained in other foods.	II a	B

#### 3.11.1. Observational Studies

The epidemiologic evidence relating egg-consumption to coronary disease risk is not entirely consistent. Most large population studies did not find an association between egg consumption and CVD [93,94,95]. However, data from 20,000 men over 20 years follow up in the Physicians’ Health Study have shown that egg consumption of at least 7 per week was associated with an increased risk of heart failure (HF). Compared with subjects who reported egg consumption of <1 per week, hazard ratios for HF were 1.28 (95% CI 1.02–1.61) and 1.64 (95% CI 1.08–2.49) for egg consumption of 1 per day and ≥2 per day, respectively [96]. Although egg consumption was not associated with incident MI or stroke in a multivariate Cox regression in this study, adjusted HRs (95% CI) for mortality were 1.0 (reference group), 0.94 (0.87–1.02), 1.03 (0.95–1.11), 1.05 (0.93–1.19), and 1.23 (1.11–1.36) for the consumption of <1, 1, 2–4, 5–6, and ≥7 eggs/week, respectively (*p* for trend < 0.0001) [97]. In several studies, consumption of at least 5 eggs per week was associated with CVD and mortality in people with diabetes [98].

#### 3.11.2. Intervention Studies

In a meta-analysis of 17 intervention studies lasting at least 14 days, the addition of 100 mg dietary cholesterol per day increased cholesterol levels by 2.2 mg/dL, while HDL-C also increased by 0.3 mg/dL [99].

#### 3.11.3. Biological Mechanisms

There is a great variation in the response of blood cholesterol levels to dietary cholesterol, possibly related to the large variability in intestinal absorption of cholesterol. It is also possible that the fat composition of eggs (high MUFA and lower SFA) restrains the blood LDL-C elevation [100].

## 4. Nutritional Supplements

### 4.1. Salt and Sodium

A low-sodium diet fits all dietary strategies. Dietary sources for sodium include: table salt, soups and gravies, soy and other sauces, salad dressing, industrially prepared meat (such as salami or industrialized frozen meat), cheese, snacks such as pretzels and popcorn, pickled foods and industrialized food in general (health services information). On average, as dietary salt (sodium chloride) intake rises, so does BP. Evidence includes results from animal studies, epidemiological studies, clinical trials, and meta-analyses of trials. In a meta-analysis including a total of 17 trials in hypertensives (*n* = 734) and 11 trials in normotensives (*n* = 2220), a median reduction in urinary sodium of ≈1.8 g/day lowered systolic BP and diastolic BP by 2.0 and 1.0 mmHg in nonhypertensive and by 5.0 and 2.7 mmHg in hypertensive individuals [101]. A recent Cochrane database review summarized three studies in normotensives (*n* = 3518), two in hypertensives (*n* = 758), one in a mixed population of normo- and hypertensives (*n* = 1981), and one in heart failure (*n* = 232) with end of trial follow-up of seven to 36 months and longest observational follow up (after trial end) of 12.7 years. Reduction of salt intake was not associated with CVD morbidity or all-cause mortality in general, and paradoxically increased the risk of all-cause death in those with congestive heart failure (end of trial RR = 2.59, 95% CI 1.04–6.44, 21 deaths) [102]. Despite these results, the authors concluded that the sample size had insufficient power to exclude clinically important effects of reduced dietary salt on mortality or CV morbidity in normotensive or hypertensive populations. Recently, the Institute of Medicine committee concluded that, although sodium restriction is recommended, evidence from studies on direct health outcomes is inconsistent and insufficient to conclude that lowering sodium intakes below 2300 mg per day either increases or decreases risk of CVD outcomes (including stroke and CVD mortality) or all-cause mortality in the general U.S. population [103].

### 4.2. Antioxidant Vitamins E and C

While being supported by observational studies, randomized controlled trials have not supported a role for vitamins in the primary or secondary prevention of CVD, and have in some cases even indicated increased mortality in those with pre-existing late-stage atherosclerosis. In a meta-analysis of 56 trials with a low risk of bias, the antioxidant supplements modestly increased mortality (RR = 1.04, 95% CI 1.01–1.07). In intervention trials including vitamins A, C, E, beta-carotene, and selenium, no beneficial effect was detected on all cause mortality in secondary prevention. Vitamin A, beta-carotene, and vitamin E supplementation increased total mortality (RR = 1.06, CI 95% 1.04–1.10) [104].

Studies have also indicated that beta-carotene mediates pro-oxidant effects. The trials that used a combination of vitamins that include beta-carotene have been disappointing. Studies also suggest that vitamins would be beneficial to individuals who are antioxidant-deficient [105]. A recent trial reported that consumption of a multivitamin had no effect on CVD risk in men [106].

### 4.3. Vitamin D

The association between vitamin D and bone disease is well established. However, vitamin D has many other functions and the use of vitamin D supplements to prevent and treat a wide range of illnesses has increased substantially over the last decade. Epidemiologic evidence links vitamin D deficiency to autoimmune disease, cancer, CVD, depression, dementia, infectious diseases, musculoskeletal decline, and more [107].

A diet high in oily fish prevents vitamin D deficiency. Solar ultraviolet B radiation penetrates the skin and converts 7-dehydrocholesterol to pre-vitamin D3, which is rapidly converted to vitamin D3 [108]. Fortified milk with vitamin D is also a source for vitamin D.

#### 4.3.1. Observation Studies

In a meta-analysis of five prospective cohort studies, the RR for CV events was 1.34 (95% CI 1.08–1.67) during an average follow up of 11.8 years [109]. A meta-analysis supported an overall association of 25-OH-D baseline levels in the lowest compared to the highest categories, with CV events (pooled HR = 1.54, 95% CI 1.22–1.95) [110]. A meta-analysis of 90 prospective studies demonstrated a linear inverse association between blood 25-(OH)-vitamin D ranging 20–60 nM/L and CVD risk [111].

#### 4.3.2. Randomized Trials

In a meta-analysis of osteoporosis intervention trials, four trials (in five articles) reported the effect of vitamin D supplementation on incident CVD. None reported a statistically significant effect of vitamin D supplementation (with or without calcium) on myocardial infarction, stroke, and other cardiac and cerebrovascular outcomes. Study participants were followed for 1, 5, or 7 years. The Women’s Health Initiative trial performed 12 analyses of different CV outcomes, and reported a near statistically significant harmful effect with combined vitamin D and calcium supplementation on one composite cardiac outcome that included non-fatal myocardial infarction, coronary heart disease death, or need for revascularization (RR = 1.08; 95% CI 0.99–1.19) [112].

In summary, at this time no recommendations can be made for vitamin D screening or treatment in populations without risk for bone fractures, for the sake of preventing CVD. Further investigation is needed to find whether treatment for vitamin D deficiency can reduce CVD morbidity and mortality.

### 4.4. Coenzyme Q10

Coenzyme Q10 (CoQ10) is a naturally occurring, fat-soluble quinone that is localized in hydrophobic portions of cellular membranes and acts as an electron carrier in the mitochondrial respiratory chain [113]. It also functions as an antioxidant, scavenging free radicals and inhibiting lipid peroxidation [114].

Clinical studies have focused on three potential effects of CoQ10 supplementation: congestive heart failure, hypertension (HTN), and myopathy related to statin therapy.

In different CVDs, including cardiomyopathy, relatively low levels of CoQ10 in myocardial tissue have been reported. However, in a sub-analysis of 1191 patients with ischemic systolic heart failure enrolled in the CORONA study, rosuvastatin reduced CoQ10, but even in patients with a low baseline CoQ10, rosuvastatin treatment was not associated with a significantly worse outcome [115].

#### Intervention Studies

Favorable short-term clinical and hemodynamic effects of oral CoQ10 supplementation have been observed in double-blind trials, especially in people with HTN and chronic heart failure. There have been no important adverse effects reported from experiments using daily supplements of up to 200 mg CoQ10 for 6–12 months and 100 mg daily for up to 6 years [116].

In a meta-analysis of 12 trials, ejection fraction was evaluated in 10 studies (*n* = 277) and cardiac output in two studies (*n* = 42). Doses ranged from 60 to 200 mg/day with treatment periods ranging from 1 to 6 months. There was a 3.7% net improvement in ejection fraction [117]. However, the long-term effect of this supplementation on clinical outcome is unknown.

In a meta-analysis of five trials including 194 patients, treatment with coenzyme Q10 significantly improved endothelial function as assessed peripherally by flow-mediated dilatation (SMD 1.70, 95% CI: 1.00–2.4, *p* < 0.0001). However, the endothelial function assessed peripherally by nitrate-mediated arterial dilatation was not significantly improved [118].

In a meta-analysis of three trials assessing treatment with CoQ10 in subjects with systolic BP > 140 mmHg and diastolic BP > 90 mmHg, there was a significant reduction of 11 (95% CI 8–14) mmHg and 7 (95% CI 5–8) mmHg, respectively. However, the authors conclude that due to the possible unreliability of some of the included studies, it is uncertain whether or not CoQ10 reduces blood pressure in the long-term management of primary HTN [119].

Statins inhibit 3-hydroxy-3-methylglutaryl coenzyme A (HMG-CoA) reductase, blocking cholesterol synthesis at a step that not only reduces cholesterol synthesis but also the production of other metabolites, including ubiquinone CoQ10. Statins reduce plasma/serum levels of CoQ10 16% to 54%, mainly as a result of reducing serum LDL, which is its major transporter [120]. The effects of statins on skeletal muscle with CoQ10 supplementation were inconsistent. Supplementation of CoQ10 increases these levels [121]. However, the effect of CoQ10 supplementation on patients with statin myopathy is inconsistent, and recent randomized trials of coenzyme Q10 supplementation have shown conflicting results [121].

### 4.5. Magnesium

Magnesium (Mg) is an abundant intracellular mineral in the body. Approximately 50% of total body Mg is found in bone. Only 1% of Mg is found in serum, and it remains constant within a wide range of intake levels. Therefore, Mg status is difficult to determine from serum Mg measurements [122]. Dietary sources of Mg are green leafy vegetables (particularly spinach), nuts, avocados, whole grains, legumes (beans and peas), soy beans, chocolate, and some seafood [123]. The recommended daily intake is 420 mg/day for men and 320 mg/day for women. Maximum recommended daily intake from supplements is 350 mg/day of elemental Mg, based on Dietary Reference Intake (DRI) [123].

#### 4.5.1. Observational Studies

Observational epidemiological studies have shown that the Mg content of drinking water and food is inversely related to morbidity and mortality from heart disease and stroke [124,125,126].

The highest quartile compared with the lowest quartile of Mg daily intake (a difference of 100 mg/1000 kcal/day between highest and lowest quartiles) was associated with a significant 31% reduction of the metabolic syndrome: HR = 0.69 (95% CI 0.52–0.91; *p* for trend <0.01) [127].

#### 4.5.2. Intervention Studies

Relatively small studies have shown a distinct advantage in providing Mg versus placebo on reducing mortality in patients with acute MI; however, two major studies published in recent years have failed to prove this [128].

Intervention studies have indicated that Mg supplementation was effective in patients with heart failure receiving diuretic therapy that reduces both Mg and potassium levels [129]. Oral Mg (365–1200 mg/day for 3–6 months) improved endothelial function [130] and inhibited platelet-dependent thrombosis in patients with CAD [131].

#### 4.5.3. Conclusions

The effect of Mg on the primary and secondary prevention of CV morbidity and mortality as well as all-cause mortality remains unclear, and therefore it is not yet possible to give conclusive recommendations in this respect.

### 4.6. Homocysteine-Reducing Agents

Homocysteine is an amino acid that contains sulfur and is produced in the body during the breakdown of the amino acid methionine. Part of the homocysteine formed in this process is recycled back to build methionine, while the rest is excreted in the urine. Folic acid, vitamin B12, and vitamin B6 regulate the metabolism of homocysteine. Deficiencies of one of these vitamins can lead to high blood homocysteine level. The normal range of blood homocysteine is 5–15 mM/L [132]. Major food sources of folic acid are: chicken liver, leafy green vegetables (spinach, broccoli, lettuce, kale, Swiss chard), beans (dried lentils, chickpeas), enriched flour, citrus fruits (mainly oranges), fortified cereals, and wheat germ. Food sources for vitamin B12 include animal products: beef, chicken, fish, egg yolk; dairy products; and fortified foods (such as cereals). Women of childbearing age should consume 400 mcg/d of folic acid for the prevention of neural tube defects of the fetus [133].

#### 4.6.1. Observation Studies

In many studies, high homocysteine levels are associated with increased risk of MI and/or stroke. Since folic acid, B_12_, and B6 (separately and combined) decreased the blood homocysteine level in 20%–40%, from baseline, it has been postulated that these supplements, can subsequently reduce CVD risk [134].

#### 4.6.2. Intervention Studies

The effectiveness of folic acid and B vitamin supplementation was examined mainly in secondary prevention intervention studies. These studies failed to prove that reducing homocysteine level by folic acid and vitamin B supplements improves CVD incidence [134]. In the Norwegian Vitamin Trial (NORVIT), the RR of re-infarction incidence, stroke, or sudden death in the group receiving 0.8 mg folic acid, 0.4 mg vitamin B12, and 40 mg Vitamin B6 compared to a control group was: 1.22, 95% CI 1.00–1.50; *p* = 0.05 [135]. The effect in primary and secondary prevention of stroke was minimal, as shown in a meta-analysis of 13 trials and 39,005 participants. The risk of stroke in those taking folic acid and vitamins B12 and B6 was RR = 0.83, 95% CI 0.71–0.97 [136]. A meta-analysis of folic acid supplementation in patients with chronic kidney disease also failed to show a beneficial effect in cardiovascular outcome [137].

### 4.7. Omega-3 and Fish Oil

Polyunsaturated fatty acids are characterized according to the position of the first double bond. In omega-3 (also called ω-3 or *n*-3) fatty acids the first double bond is situated after the third carbon atom from the methyl end of the carbon chain. Humans cannot synthesize short-chain fatty acids and therefore need to consume them in their diet. They include the plant-derived alpha-linolenic acid (ALA, 18:3*n*-3), and the fish-oil-derived eicosapentaenoic acid (EPA, 20:5*n*-3) and docosahexaenoic acid (DHA, 22:6*n*-3).

#### 4.7.1. Dietary Sources

ALA is found in seeds, vegetable oils (especially canola and flaxseed), green leafy vegetables, walnuts, and beans. Although some ALA can be transformed in the human body to EPA and DHA, such conversion appears to be inefficient [138], and the majority of these fatty acids are consumed from cold water oily fish, such as salmon, herring, mackerel, anchovies, tuna, and sardines.

#### 4.7.2. Omega-3 Supplements

Various sources of omega-3 fatty acids are used as supplements for commercial use, including fish oil, flaxseed oil, and walnut oil. Although the FDA has concluded that omega-3 dietary supplements from fish are “generally recognized as safe”, some have questioned the safety of fish oil supplements because some species of fish can contain high levels of mercury, pesticides, or polychlorinated biphenyls (PCBs). Most fish oil supplements undergo purification processes and do not appear to contain these substances in appreciable quantities. Many clinical trials have used an ethyl-ester form of omega-3 fatty acids, which may affect the product’s bioavailability and metabolism [139]. Commonly used doses of omega-3 supplements (up to 1 g daily) do not appear to have significant side effects. However, larger doses may cause minor gastrointestinal upsets, worsening of glycemia control, and a rise in LDL-C levels [140].

#### 4.7.3. Observational Studies

Most observational studies show an inverse correlation between fish consumption and cardiovascular CVD. A review of 11 cohort studies involving 116,764 individuals suggested that fish consumption at 40–60 g daily is associated with markedly reduced CHD mortality in high-risk, but not in low-risk populations [141].

#### 4.7.4. Intervention Studies

A meta-analysis of intervention trials including 7951 individuals treated with omega-3 compared to 7855 controls found a significant decrease in mortality from MI but not in non-lethal MI [142]. In another meta-analysis of 97 studies using different types of lipid management strategies, the most effective combination was that of statins with omega-3, which resulted in a relative-risk reduction of 23% in total mortality (RR = 0.77, 95% CI 0.63–0.94) and 32% in cardiac mortality (RR = 0.68; 95% CI 0.52–0.90) [143]. However, more recent studies looking at the benefit of omega-3 treatment in high-risk patients (CHD and/or diabetes mellitus) receiving optimal medical therapy, including statins, have shown mixed results with some showing significant benefit [144] while others show little additional benefit [145,146,147]. Recent meta-analyses of randomized controlled trials found little evidence of a protective effect of omega-3 supplementation on the incidence of CVD [148], cerebrovascular disease [149], or atrial fibrillation [150]. In a meta-analysis of 20 studies of 68,680 patients (13 on secondary prevention), omega-3 PUFA supplementation was not associated with a lower risk of all-cause mortality, cardiac death, sudden death, myocardial infarction, or stroke based on relative and absolute measures of association. [151].

#### 4.7.5. Possible Mechanisms

The long-chain omega-3 fatty acids EPA and DHA compete with arachidonic acid (a long chain omega-6 fatty acid) in the synthesis of prostaglandins and leukotrienes involved in inflammation and thrombogenesis. Omega-3 fatty acids have been shown to increase arrhythmic thresholds, reduce blood pressure, improve endothelial function, reduce inflammation and platelet aggregation, enhance plaque stabilization, and favorably affect autonomic tone [152]. At high doses (2–6 g daily) they can significantly reduce the serum triglyceride levels, but the long-term clinical outcome of such treatment in hypertriglyceridemic individuals has not been evaluated [153].

### 4.8. Phytosterols

Sterols constitute an important constituent of plant cellular membranes, in a manner similar to the role of cholesterol in human cells [154]. They are found at low concentrations in most plant-derived nutrients but at somewhat higher concentrations in some grains. Despite their structural similarities to cholesterol, plant sterols are not synthesized in the human body and are only minimally absorbed from the human intestinal tract. The average western diet contains approximately 200–500 mg of cholesterol, approximately 200–400 mg of plant sterols, and 20–50 mg of plant stanols. Amongst the best known plant sterols are sitosterol, campesterol, and stigmasterol. Those that are incorporated in food are usually esterified. Hydrogenation converts sterols into stanols (e.g., sitostanol and campestanol), which can also be esterified.

#### 4.8.1. Intervention Studies

Evaluation of intervention studies with sterol esters and stanol esters suggest a reduction in LDL-C level of approximately 10%, without specific differences between the type of sterol or stanol or the method by which it was administered (immersed in a food product or as a separate supplement) [155,156,157,158]. Similar results were obtained in the different populations studied (children, healthy adults, or patients with diabetes and/or CHD) [159,160]. The optimal dose appears to be 1.5–2.5 g/day, with no additional benefit at higher doses [161]. Addition to statins resulted in a further 10% reduction in LDL-C beyond that achieved with the statins alone [162]. To date, no long-term intervention studies of sterol/stanol treatment evaluating clinical endpoints have been published.

#### 4.8.2. Possible Mechanisms

Due to their biochemical similarity, plant sterols and stanols can displace cholesterol from mixed micelles in the intestine, thus reducing the absorption of dietary cholesterol [163]. Although they have significant atherogenic potential, the intestinal absorption of sterols and stanols is poor, resulting in very low serum concentrations. An exception to this rule is patients with sitosterolemia, a rare genetic disorder in which the absorption of sterols is enhanced, resulting in significant damage to various organs.

Level of evidence and classes of recommendations for nutritional supplements is summarized in Table 8.

**Table 8:** Level of evidence and classes of recommendations for nutritional supplements.

Supplement	Recommendations	Strength	Level of evidence
**Sodium**	It is recommended to limit salt intake to 2.3 g sodium (6 g/day salt). It is recommended to substitute salt with other spices and herbs. It is recommended to use food labels for information of sodium content in foods.	I	B
	It is recommended to reduce as much as possible the use of industrial pre-prepared food, as well as salted snacks and vegetables.	I	B
	Efforts should be put into reducing sodium content in industrial foods through legislation.	I	B
**Omega-3**			
General population (primary prevention)	Eat a variety of fish, preferably fat, at least twice a week. Each fish portion (55–85 g) should supply at least 500–1000 mg EPA + DHA.	II a	A
	It is recommended not to exceed 200 g daily of fish that contain a high level of mercury (such as mackerel, sword fish or shark) or 400 g of other fish. Removing the skin off the fish before preparation can reduce the amount of contaminants.	I	B
	For children and pregnant women it is recommended to avoid eating fish with potentially high levels of contaminants.	III	B
	Omega-3 supplements containing 1 g of EPA + DHA.	II b	B
People with proven CVD	Individuals who do not regularly consume fish might consider ingesting omega-3 supplements containing 1 g of EPA + DHA.	II b	A
Hypertriglyceridemia	2–6 g of omega 3 daily can reduce serum TG levels. However, no long-term studies have been conducted to evaluate the clinical outcome in these individuals	II b	B
**Phytosterols**	Plant phytosterols can be considered for the reduction of LDL cholesterol in mildly hypercholesterolemic individuals at intermediate to high risk who do not wish to use, or cannot tolerate, other cholesterol-lowering medications.	II a	A
	Plant phytosterols can be used in combination with statins for additional reduction of LDL cholesterol.	II b	A
**Antioxidant-vitamin supplementation**	Based on data from intervention controlled trials, it is not recommended to use antioxidant vitamins supplementation to prevent or treat CVD.	III	A
**Vitamin D**	At this point there is no recommendation for screening blood vitamin D levels for CVD prevention.	III	C
	Correction of low vitamin D levels may reduce CVD morbidity and mortality.	II b	B
	It is not recommended to use vitamin D supplements in order to prevent CVD in people with normal vitamin D levels.	III	C
**CoQ10**	The long term effect of CoQ10 supplementation on Patients with CHF and/or treated with statins is yet to be proven. Therefore it is not recommended to use CoQ10 supplementation in these patients.	III	B
	In the short term Co Q10 supplementation results in mild blood pressure reduction and mild increase in ejection fraction in CHF patients.	II b	B
**Magnesium (Mg)**	At this point there is no recommendation for screening blood magnesium levels for CVD prevention in general population.	III	C
	Correction of low magnesium levels may reduce CVD morbidity and mortality, particularly after myocardial infarction.	II a	A
	It is not recommended to use magnesium supplements in order to prevent CVD in people with normal magnesium levels.	III	A
**Folate, vitamin B6, vitamin B12**	Low serum folate and/or vitamin B12 concentrations should be corrected to prevent neurologic and hematologic diseases. Women of childbearing age should consume 400 µg/day of folic acid supplementation for the prevention of neural tube defects of the fetus.	I	A
	Folic acid and vitamin B supplements are not effective for primary, nor for secondary prevention of CVD and stroke.	III	A

#### 4.8.3. Safety

Sterol supplementation at the recommended doses is generally considered safe [164]. However, several potential risks need to be considered. In addition to inhibiting cholesterol absorption, some (though not all) studies suggest that sterols and stanols can reduce the blood levels of antioxidants such as lycopene and beta-carotene. [157,165]. This can be counteracted, at least partly, by the ingestion of a diet reach in vegetables and fruits [166]. Despite the low serum concentration of sterols and stanols, some concern has been raised that even the slight increase associated with dietary supplementation of sterols might increase the risk for atherosclerosis [167].

## 5. Conclusions

A healthy diet should include diversity of foods and to maintain a healthy weight. It is preferable to eat fresh or frozen food without additional sugar, salt or high-calorie gravies, using cooking methods that retain the original nutrients undestroyed. It should contain a variety of vegetables and fruits, legumes, whole grains, whole wheat bread and high-fiber low-salt food items. Vegetable oils, (especially olive and canola oils, excluding palm and coconut oils), should be preferred over animal fat. Additional elements that may confer health benefits include avocado, nuts, almonds and tahini, low-fat dairy products, green tea and 2 to 3 servings of fatty fish per week. It is recommended to minimize consumption of high-fat meat (especially processed meats that are high in fat and sodium), hard margarines and pastries with hydrogenated fat, and foods that are high in sodium and sugar. It is recommended to drink a lot of water, and reduce consumption of sweetened beverages as well as fresh juices. The Mediterranean diet has been shown to reduce cardiovascular morbidity and mortality in both primary and secondary prevention. Other dietary patterns that have been shown to confer advantage in specific medical situations include low-fat diet for individuals at high cardiovascular risk, DASH diet for people with hypertension, and low-carbohydrate diets for overweight people and for the metabolic syndrome.
